# Supplementary material for: Predicting human decision making in psychological tasks with recurrent neural networks
Source: PLoS One. 2022 May 31;17(5):e0267907. doi: 10.1371/journal.pone.0267907 (PMC9154096; doi:10.1371/journal.pone.0267907)
Supplement: Attachment — Submitted filename: response_HumanLSTM.pdf [file pone.0267907.s001.pdf]

RESPONSES TO THE REVIEWER

We thank the reviewer for the careful read and constructive feedback. As you can see from our difference-highlighted revision manuscript, we have fully taken into account your helpful suggestions and significantly revised our manuscript. The changes are highlighted in detail in the attached file (difference_HumanLSTM.pdf) and summarized as follows:

- We modified the abstract to reflect a more explicit, constrained and solid description of what we have accomplished.
- To better address the significance of our idea, we modified the last paragraph of the introduction to address the main objectives.
- The structure of the paper is now reorganized in two ways: we merged the “Background” into the “Materials and Methods”, and separate the “Discussion” out of the “Results”, per your suggestion.
- We changed the description of the tables to distinguish it from the main text.
- We introduced more details about the data collection for both the Iowa Gambling Task and Iterated Prisoner’s Dilemma into the “Methods and Materials” section.
- The description of the recurrent neural network was moved forward, because it is the main methodological component.
- We significantly rewrote our “Discussion” section such that core conclusions are well structured and connected to the conclusions. Main contributions are reiterated in the front and the back. The interpretations related to individual experiments are discussed in more details.

We hope these improvements in the writing are able to better address the significance of our idea. We thank you for your time and efforts again for reviewing our manuscript.
